# Identification and distribution of a downregulatory signaling alkyloxazole in *Streptomyces*

**DOI:** 10.1073/pnas.2600233123

**Published:** 2026-07-06

**Authors:** Michael Madden, Dan Xue, Mingming Xu, Katherine Holandez-Lopez, Joseph Budiselich, Sarah Tran, Cole Espinosa, Jie Li

**Affiliations:** ^a^ Division of Chemical Biology and Medicinal Chemistry, College of Pharmacy, University of Texas at Austin, Austin, TX 78712; ^b^ Department of Chemistry and Biochemistry, University of South Carolina, Columbia, SC 29208

**Keywords:** signaling molecule, *Streptomyces*, biosynthesis, genome mining

## Abstract

*Streptomyces* use small molecules to dictate morphological development and secondary metabolism. Most of these signaling compounds are inducers derived from γ-butyrolactone (GBL)-synthesizing enzymes. As such, biosynthetically distinct and downregulatory signaling molecules remain understudied in *Streptomyces*. Here, we report the identification of alkyloxaozle (AOX, **1**), a downregulator of sporulation and antibiotic production in *Streptomyces*. **1**’s structure features an oxazole head flanked by an alkyl tail synthesized by a nonribosomal peptide synthetase (NRPS) that has not been reported in *Streptomyces* signaling molecules. Functional studies found that **1** downregulated sporulation and secondary metabolism in several *Streptomyces* strains. Genome mining identified 45 homologous biosynthetic gene clusters (BGCs) only distributed in *Streptomyces*, underscoring the importance of AOXs to this productive genus. Altogether, this report presents a downregulatory class of signaling molecules that broadly affect *Streptomyces* and highlights their importance to the genus.

*Streptomyces* is a model bacterial genus for studying microbial physiology and the production of drugs widely used in clinics (1). These life processes in *Streptomyces* are mediated by small molecules—best represented by γ-butryolactones (GBLs), butenolides, and methylenomycin furans (MMFs) (2–4). While these positive regulatory inducers are well documented, inhibitory downregulators remain underexplored in this genus. Furthermore, most signaling compounds were identified from homology searches of the GBL-producing AfsA (2–4). This narrow scope limits the discovery of functionally and structurally distinct signaling molecules. Herein, we report a downregulatory signaling alkyloxazole (AOX, **1**) isolated from *Streptomyces davaonensis* DSM 101723. **1**’s downregulation in multiple strains and distinct biosynthetic origin expand the scope of small molecule regulation in *Streptomyces*.

## Results

**Fig. 1.**
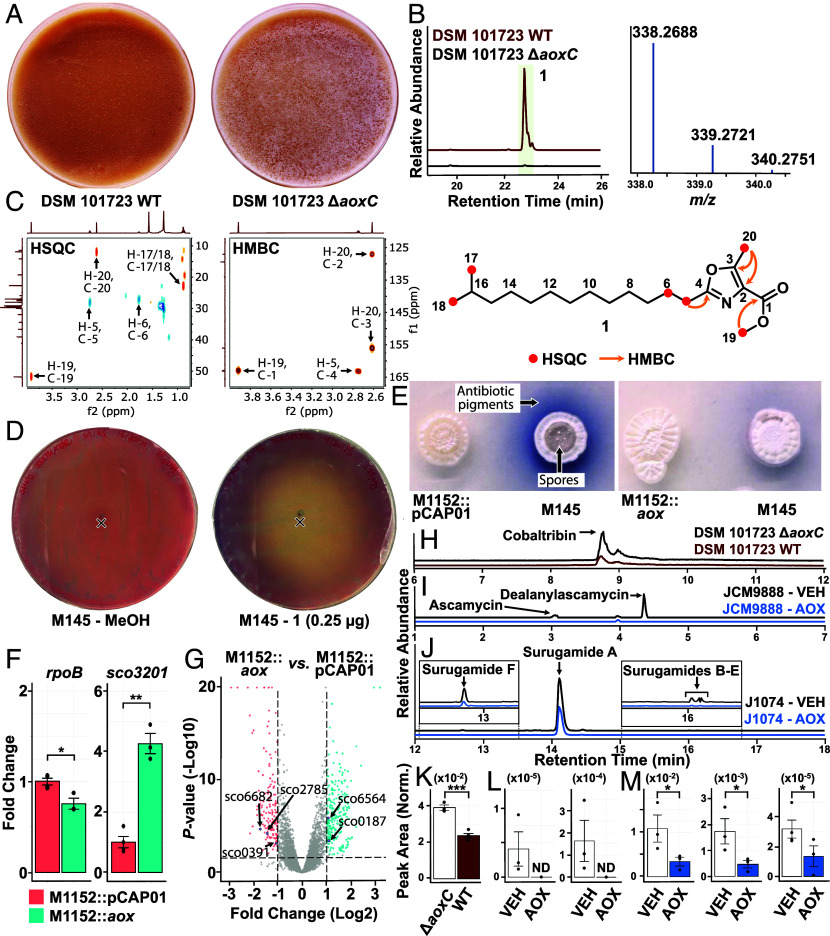
Characterization of **1** and its downregulatory activity. (*A*) Sporulation plates of *S. davaonensis* DSM 101723 wild type (DSM 101723 WT) and Δ*aoxC* mutant after 5 d, suggesting *aoxC*’s product as a sporulation downregulator. (*B*) Extracted ion chromatograms (EICs) of DSM 101723 WT and Δ*aoxC* mutant extracts (*Left*) and the mass spectrum of **1** (*Right*). (*C*) Representative portions of HSQC (*Left*) and HMBC (*Middle*) NMR spectra and the representation of correlations of **1** (*Right*). (*D*) M145 lawns on SMMS treated with a methanol vehicle (*Left*) or 0.25 μg of **1** (*Right*) after 51 h showing decreased pigment production around the application site, indicated by “X”. (*E*) M145 coculture assays with M1152::pCAP01 (*Left*) and M1152::*aox* (*Right*) on SMMS, showing decreased sporulation and pigment production with M1152::*aox* after 10 d. (*F*) RT-qPCR analysis of *rpoB* (*Left*) and *sco3201* (*Right*) showing differential gene expressions in M1152::*aox*, with significance determined using a two-sided Student’s *t* test. (*G*) Volcano plot of differentially expressed genes between M1152::*aox* and M1152::pCAP01, with genes associated with *rpoB* and *sco3201* pathways highlighted. (*H–J*) EICs highlighting differential productions of cobaltribin between DSM 101723 Δa*oxC* and WT (*H*), ascamycins between vehicle (VEH) and AOX-treated *S.* sp. JCM9888 (*I*), and surugamides between VEH and AOX-treated *S. albus* J1074 (*J*). (*K–M*) Quantification of cobaltribin in DSM 101723 Δ*aoxC* and WT (*K*), ascamycin (*Left*), and dealanylascamycin (*Right*) in VEH and AOX-treated *S.* sp. JCM9888 (*L*), as well as surugamides A (*Left*), B-E (*Middle*), and F (*Right*) in VEH and AOX-treated *S. albus* J1074 (*M*), with significance determined using a one-sided Student’s *t* test. Each bar represents the mean ± SEM of 3 to 4 biological replicates; *P ≤ 0.05, **P ≤ 0.01, ***P ≤ 0.001, ND = not detected.

While characterizing secondary metabolites from *S. davaonensis* DSM 101723 (5, 6), we generated a series of genetic mutants. Knocking out *aoxC* enhanced sporulation in the resulting mutant (Δ*aoxC*, Fig. 1*A*). As small molecules regulate morphological differentiation, we hypothesized that *aoxC* may be involved in the biosynthesis of a signaling molecule. Comparative liquid chromatography-mass spectrometry (LC-MS) analysis of wild-type (WT) and Δ*aoxC* mutant extracts revealed a metabolite associated with *aoxC* (**1**, Fig. 1*B*). Extracellular **1** reached a maximum concentration of 18.9 ng/μL during the stationary phase [*SI Appendix*, Fig. S1 (7)]. We next identified **1**’s biosynthetic gene cluster (BGC)*, aox*, and integrated it into an *S. coelicolor* M1152 heterologous overexpression host to enhance **1**’s production. The generated strain, M1152::*aox*, was cultured then **1** was isolated and characterized as described in the *SI Appendix*, *Extended Methods* and Dataset S1. Altogether, **1** was identified as an alkyloxazole (AOX) decorated with a methyl ester group (Fig. 1*C*).

We next verified **1**’s downregulatory function. We first used a bioindicator strain, *S. coelicolor* M145 (M145). Signaling molecules affect the production of the pigmented antibiotics actinorhodin (Act; blue) and undecylprodigiosin (Red; red) in M145, enabling the verification of signaling by easily visible color changes (8). Treatment with 0.25 μg of **1** produced less pigment near the application site compared to the methanol vehicle (Fig. 1*D*), indicating downregulated antibiotic production. To show **1**’s downregulatory signaling in vivo, M145 was cocultured 2 cm from M1152::*aox* or an expression control strain harboring an empty plasmid, M1152::pCAP01. M145 cocultured with M1152::*aox* exhibited less sporulation and pigment production (Fig. 1*E*), establishing **1**’s downregulation of secondary metabolism and morphological differentiation. To explore **1**’s potential signaling pathways, we compared gene expression between M1152::pCAP01 and M1152::*aox*. We analyzed genes connected to morphological differentiation and antibiotic production or have been less-studied in *Streptomyces*. Reverse transcriptase-quantitative PCR (RT-qPCR) found decreased *rpoB* and increased *sco3201* expressions in M1152::*aox* (Fig. 1*F* and Dataset S2). RpoB is involved in the activation of secondary metabolism (9) while SCO3201, an orphan transcriptional regulator (10), inhibits secondary metabolism and sporulation upon overexpression. Thus, **1**’s downregulatory activity may partially arise from suppressing *rpoB* while activating *sco3201*. Notably, *scbA* expression and GBL production were not altered in M1152::*aox* [*SI Appendix*, Fig. S2 (11)]. RNA-Seq analysis of M1152::*aox vs.*M1152::pCAP01 identified additional differently expressed genes, including those associated with the signaling pathways of *sco3201* (*sco0187*, *sco6564*, and *sco6682*), *rpoB* (*sco0391*), or both (*sco2785*) (Fig. 1*G* and Dataset S3) (9, 12). We then expanded **1**’s downregulation to additional metabolites in multiple *Streptomyces* species (Fig. 1 *H*–*M* and Dataset S4). Compared to the Δ*aoxC* mutant, DSM 101723 WT produced less cobaltribin (6). Similarly, adding 2.5 µg of **1** in 10 mL liquid cultures ablated ascamycin (13) production in *S.* sp. JCM9888 and decreased surugamide (14) production in *S. albus* J1074, highlighting **1**’s effects beyond its native producer.

**Fig. 2.**
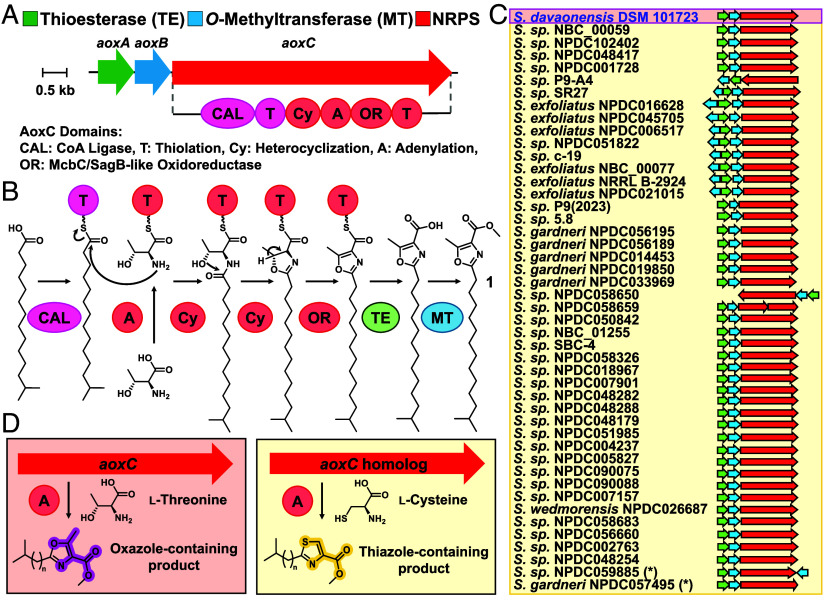
Bioinformatic interrogation of **1**’s biosynthetic distribution. (*A*) Annotation of the *aox* BGC. (*B*) Proposed biosynthetic scheme of **1**. (*C*) Strains with *aox* homologs and their genetic organizations color-coded by the predicted products corresponding to panel *D*. (*) indicates BGCs whose A-domain substrates could not be predicted by antiSMASH and were instead predicted by PARAS. (*D*) Predicted products of *aox* and its homologs, incorporating threonine (purple) or cysteine (yellow).

Inspired by **1**’s structure and effect in multiple strains, we investigated its biosynthesis and distribution. AntiSMASH 8.0 analysis found that *aox* was made up of three genes encoding a thioesterase (*aoxA*), an *O*-methyltransferase (*aoxB*), and a nonribosomal peptide synthetase (NRPS, *aoxC*) (Fig. 2*A*). From these genes, we proposed **1**’s biosynthesis as depicted in Fig. 2*B* and explained in the *SI Appendix*, *Extended Methods*. Due to AoxC’s importance in synthesizing **1**’s scaffold, we used it to mine for other AOX-producing strains. Our search yielded 45 homologous BGCs (Fig. 2*C*). These BGCs, categorized into two groups based on their predicted substrates (threonine or cysteine) (Fig. 2*D*), were only found in *Streptomyces*, suggesting important and conserved signaling functions of AOXs in this genus.

## Discussion

The downregulation of both sporulation and antibiotic production by **1** is rare among *Streptomyces* signaling molecules, as only chalcone has been reported to inhibit both processes (15). However, the studies with chalcone were only conducted in M145, making **1**’s ability to downregulate across multiple strains a remarkable expansion. **1**’s oxazole head group has not been reported in *Streptomyces* signaling molecules, positioning **1** as the founding member of another class of signaling compounds in the genus. The increased expression of *sco3201* in M1152::*aox* was intriguing as SCO3201 is an orphan transcriptional regulator (10), warranting further studies into **1**’s potential as a ligand for SCO3201. As most *Streptomyces* signaling molecules were discovered through mining for AfsA homologs, **1**’s non-AfsA origin highlights the underexplored potential of non-AfsA-derived signaling molecules. Given the prevalence of *aox* variants and silent biosynthetic genes across *Streptomyces*, targeting these BGCs through genetic engineering may enhance secondary metabolite discovery (16). Most of the homologous BGCs we identified were predicted to incorporate cysteine instead of threonine to form thiazole-featuring analogs, suggesting further chemical diversity within AOXs (Fig. 2 *C* and *D*). Overall, our discovery of **1** expands the known regulatory roles and biosynthetic origins of *Streptomyces* signaling molecules.

## Materials and Methods

Sporulation assays were conducted on mannitol soy flour (MS) agar. M145 antibiotic production and coculture assays were conducted on Supplemented Minimal Media Solid (SMMS), based on previous works (8, 17). Metabolite production was analyzed and quantified by HR-ESI-MS. Details for general procedures, culture conditions, gene editing, compound isolation, structure elucidation, gene expression analysis, and genome mining are found in the *SI Appendix*.

## Supplementary Material

Appendix 01 (PDF)

Dataset S01 (XLSX)

Dataset S02 (XLSX)

Dataset S03 (XLSX)

Dataset S04 (XLSX)

## Data Availability

Supplementary figures data have been deposited in Figshare (https://doi.org/10.6084/m9.figshare.32563290, https://doi.org/10.6084/m9.figshare.32564454) (7, 11). Study data are included in the article and/or supporting information.
