# Supplementary material for: Identification and distribution of a downregulatory signaling alkyloxazole in Streptomyces
Source: Proc Natl Acad Sci U S A. 2026 Jul 6;123(28):e2600233123. doi: 10.1073/pnas.2600233123 (PMC13367870; doi:10.1073/pnas.2600233123)
Supplement: Supplementary file 1 — Appendix 01 (PDF) [file pnas.2600233123.sapp.pdf]

PNAS

Supporting Information for
Identification and distribution of a downregulatory signaling alkyloxazole in *Streptomyces*

Michael Madden, Dan Xue, Mingming Xu, Katherine Holandez-Lopez, Joseph Budiselich, Sarah Tran, Cole Espinosa, Jie Li

Jie Li
Email: jie.li@austin.utexas.edu

This PDF file includes:

Supporting text
Legends for Datasets S1 to S4
SI References

Other supporting materials for this manuscript include the following:

Datasets S1 to S4

Supporting Information Text

Extended Methods

General Experimental Procedures. NMR spectra were acquired on a Bruker Advance III HD Ascend 800 MHz spectrometer equipped with a 5 mm triple-resonance Observe (TXO) cryoprobe with *Z*-gradients, controlled by TopSpin 3.6.1 software. All spectra were acquired at 25 °C in $CDCl_3$, with reference to residual ^1H or ^{13}C signals. HR-ESI-MS spectra were obtained following high-performance liquid chromatography on a Thermo Scientific Vanquish UHPLC by a Thermo Scientific Orbitrap Velos Pro hybrid ion trap–orbitrap mass spectrometer. LC-PDA-ESI-MS data were acquired on a Thermo Dionex Ultimate 3000 UHPLC system equipped with a diode array multiple wavelength detector and an LTQ XL linear ion trap mass spectrometer controlled by Thermo Xcalibur 4.2.47 software. The ion trap mass spectrometer was set at a sheath gas flow rate of 35 arbitrary units, a source heater temperature of 325 °C, and a capillary temperature of 350 °C. Thin-layer chromatography (TLC) was conducted using aluminum-backed silica gel plates with a fluorescent indicator.

Strains and Culture Conditions. *Streptomyces davaonensis* DSM 101723 was purchased from the Leibniz Institute DSMZ. *Streptomyces coelicolor* M1152 was used as a heterologous host. *Escherichia coli* ET12567 and ETZ were used for conjugation to facilitate heterologous expression and gene deletion, respectively. *Streptomyces coelicolor* M145 was used as a bioindicator strain for antibiotic production. *Streptomyces* sp. JCM9888 and *Streptomyces albus* J1074 were used as additional strains to examine secondary metabolite production potentially affected by the alkyloxazole signaling molecule (**1**). All *Streptomyces* strains were routinely cultivated for single colonies on mannitol soy flour (MS) agar with nalidixic acid (25 µg mL^{-1}) (1). *Streptomyces* spore suspensions were prepared from confluent lawns on MS plates as previously described (1). Sporulation assays were performed on MS agar without antibiotics. For large-scale cultivation of *Streptomyces* strains, single colonies were precultivated in BD Bacto trypticase soy broth (TSB) containing nalidixic acid (25 µg ml^{-1}) at 30 °C for 3 days. The pre-culture was diluted 100-fold into Streptomycete Production Medium (SPM) (2) and incubated with agitation at 30 °C for 7 days. *S. coelicolor* M145 antibiotic production and co-culture assays were performed on Supplemented Minimal Medium Solid (SMMS) (3). *E. coli* strains were routinely cultivated in Lysogeny Broth (LB) with appropriate antibiotics.

Double Cross-over Allelic Exchange-based Gene Deletion. *S. davaonensis* DSM 101723 was cultured in 5 mL TSB and genomic DNA was extracted using standard methods. Oligonucleotide primers containing appropriate overhangs were used to amplify homology arms flanking *aoxC* from *S. davaonensis* DSM 101723 genomic DNA via PCR [armL-Fwd: TGACGCCTCCCATGGTA TAAATAGTGGCTGGAGATGGGCCGCACCACA, armL-Rev: TCTAGAGAATAGGAACTTCCCA GCCCGACCTGTGTTCATGCTGTTCCTCCGGT, armR-Fwd: AGTATAGGAACTTCGAAGCAGC TCCAGCCTACACACATCCGTACGTGTCACACT, armR-Rev: AGTCCTCTTCCAACAATAATAAT GTCAGATCCTGTTGTCGTCCTCGTCGGTGTGCT]. An apramycin resistance gene (*aprR*) containing appropriate overhangs was similarly amplified via PCR from the vector pMXT19 [APR-Fwd: GGT CGGGCTGGGAAGTTCCT, APR-Rev: GTAGGCTGGAGCTGCTTCG]. The vector pCAP04 was linearized via digestion with XhoI and BsaI. The PCR products and linearized pCAP04 were then assembled into a plasmid containing *aprR* flanked by the *aoxC* homology arms via Gibson Assembly, yielding pCAP04::*aoxC*. The assembled pCAP04::*aoxC* was subsequently transformed into the methylation-deficient *E. coli* ETZ for conjugative transfer. Conjugation was then performed using *E. coli* ETZ_pCAP04::*aoxC* and *S. davaonensis* DSM 101723. *E. coli* ETZ_ ETZ_pCAP04::aoxC was cultured overnight at 37 ºC in 10 mL LB supplemented with chloramphenicol (25 µg/mL), kanamycin (50 µg/mL), and apramycin (25 µg/mL) while *S. davaonensis* DSM 101723 was cultured for 48 hr at 30 ºC in 10 mL TSB supplemented with nalidixic acid (25 µg/mL). Each culture was washed twice in 10 mL of its corresponding antibiotic-free medium, and the OD_{600} of each culture was measured. Each culture was concentrated to an OD_{600} of 5.0 by centrifugation and resuspended in its corresponding antibiotic-free medium, and 200 µL of each concentrated culture was mixed and then spread on MS agar supplemented with 10 mM $MgCl_2$, and this plate was cultured at 30 ºC for 20 hours. The nalidixic acid and apramycin were overlayed onto the plate before incubation at 30 ºC for 5 days. After incubation, exconjugants were selected and replica plated onto MS agar supplemented with nalidixic acid, kanamycin, and apramycin as well as onto MS agar supplemented with nalidixic

acid and apramycin. The replica plates were cultured at 30 ºC for three days, and colonies that grew on MS agar supplemented with nalidixic acid and apramycin, but not on MS agar supplemented with nalidixic acid, kanamycin, and apramycin, were selected as *aoxC* knockout mutants (Δ*aoxC*).

Heterologous Expression. The integrative vector pCAP01 was linearized by digestion with SpeI and XhoI to yield L-pCAP01. *S. davaonensis* DSM 101723 was cultured in 5 ml TSB and genomic DNA was extracted using standard methods. The *aox* BGC was amplified by PCR with two homology arms with L-pCAP01 and then ligated by Gibson Assembly, forming pCAP01::*aox*. Then, pCAP01::*aox* was first transformed into *E. coli* ET12567 and subsequently conjugated into *S. coelicolor* M1152 by triparental intergenic conjugation facilitated by *E. coli* ET12567/pUB307 to yield the heterologous expression host M1152::*aox*. A similar process using just pCAP01 was conducted to generate the heterologous expression control strain M1152::pCAP01.

Isolation of 1. Mature M1152::*aox* cultures in SPM were centrifuged at 6000 x*g* for 10 min at 4 °C, and the supernatant was decanted. The remaining pellet was extracted twice with ethyl acetate, dried, and lyophilized. The resulting extract was dissolved in hexane and defatted with an equal volume of methanol supplemented with 8% water. The methanol/water layer was removed, dried, and lyophilized. The defatted extract was separated using silica gel chromatography with dichloromethane and methanol. Fractions containing **1**, based on LC-PDA-ESI-MS and TLC, were repeatedly pooled and chromatographed until **1** was the only spot visible by TLC. Residual plastics from the column were removed using preparative TLC with dichloromethane and 1% methanol. The silica containing **1** was scraped from the plate and extracted with 100% ethyl acetate.

Structure Elucidation of 1. Compound **1** was isolated as a white, amorphous solid. One- and two-dimensional NMR, including ^{1}H, ^{13}C, HSQC, and HMBC, were used to elucidate the chemical structure of **1** (Figure 1C and Dataset S1). Assignment data and spectra are available in Dataset S1. ^{1}H-NMR analysis identified resonances corresponding to four methyl groups H_3-20 (δ_H 2.59, 3H, s), H_3-17 and H_3-18 (δ_H 0.86, 6H, d, J = 6.6 Hz), as well as a methoxy group H_3-19 (δ_H 3.89, 3H, s); a methine group H-16 (δ_H 1.51, 1H, m); and a series of methylenes including H_2-5 (δ_H 2.72, 2H, t, J = 7.5 Hz), H_2-6 (δ_H 1.74, 2H, m), H_2-15 (δ_H 1.15, 2H, m) and H_2-7 through H_2-14 (δ_H 1.25, 16H, m). A combination of ^{13}C-NMR and HSQC analyses identified resonances corresponding to a carbonyl C-1 (δ_C 163.3); three sp^2 carbons C-2 (δ_C 127.0), C-3 (δ_C 156.1), and C-4 (δ_C 163.0); an sp^3 methine C-16 (δ_C 29.3); four methyls C-20 (δ_C 12.0), C-17 (δ_C 22.8), C-18 (δ_C 22.8), and C-19 (δ_C 51.8); and eleven sp^3 methylenes C-5 (32.1), C-6 through C-14 (δ_C 27.1-30.1), and C-15 (δ_C 39.2). The oxazole core was identified based on the close resemblance of the sp^2 carbon signals to those from thiazole-containing natural products and synthetic (methyl)oxazoles (2, 4). HMBC correlations between H_3-20 (δ_H 2.59) and C-2 (δ_C 127.0) as well as C-3 (δ_C 156.1) positioned the methyl group on the oxazole ring at C-3. A similar HMBC correlation between H_2-5 (δ_H 2.72) and C-4 (δ_C 163.0) identified the methylene series as an aliphatic chain connected to C-4 of the oxazole ring. The integration and splitting pattern of the upfield methyl protons (δ_H 0.86) suggested that this aliphatic chain was derived from an iso-branched fatty acid. Finally, a key HMBC correlation between the methoxy H_3-19 (δ_H 3.89) and the carbonyl C-1 (δ_C 163.3) identified these signals as originating from a methyl ester group. This group's position on the oxazole was inferred based on the remaining free carbon on the heterocycle and the spectrum's resemblance to a methyl ester-bound thiazole-containing natural product (2).

__Compound 1__

^{1}H NMR (700 MHz, $CDCl_3$): δ 3.89 (s, 3H), 2.72 (t, J = 7.5 Hz, 2H), 2.59 (s, 3H), 1.74 (m, 2H), 1.51 (m, 1H), 1.25 (m, 16H), 1.15 (m, 2H), 0.86 (d, J = 6.6 Hz, 6H); ^{13}C NMR (175 MHz, $CDCl_3$): δ 163.3, 163.0, 156.1, 127.0, 51.8, 39.2, 32.1, 27.1 - 30.1 (9C), 29.3, 22.8 (2C), 12.0. HR-ESI-MS (*m/z*): $[M + H]^+$ calc'd. for $C_{20}H_{36}NO_3$, 338.2695; found, 338.2688.

DSM 101723 Growth and 1 Production Curves. A 500 mL culture of *S. davaonensis* DSM 101723 was prepared in SPM and cultured as described above. At the specified timepoints, three 1 mL aliquots of culture were transferred into pre-weighed tubes and centrifuged at 6000 x*g* for 10 min at 4 °C. The supernatant was transferred to another container and reserved for extracellular **1** quantification. The remaining cell pellet was washed three times with water, frozen, and lyophilized. The resulting cell matter was then weighed to obtain the dried cell weights. The

reserved supernatant was extracted with an equal volume of ethyl acetate, dried, and redissolved in 50 µL of methanol for HR-ESI-MS analysis. The resulting peak areas of **1** were quantified using an external standard curve prepared from the isolated **1** prepared by serial dilutions in methanol. The growth and production curves are reported in Fig. S1 on Figshare (https://doi.org/10.6084/m9.figshare.32563290) (5).

***Streptomyces* Sporulation Assay.** Confluent lawns of *S. davaonensis* DSM 101723 WT and Δ*aoxC* spores (5 x 10^6) were spread on MS agar using water and left open to dry in a biosafety cabinet. The dried plates were sealed with ParaFilm, incubated at 30°C, and monitored daily for sporulation.

***S. coelicolor* M145 Antibiotic Bioassay.** A detailed protocol has been previously published (6, 7). In short, confluent lawns of *S. coelicolor* M145 spores (5 x 10^6) were spread on SMMS and left to dry in a biosafety cabinet. 0.125 µg/µL of **1** was prepared in methanol, and 2 µL (0.25 µg) was pipetted onto the center of the plate. A control plate of M145 spores was treated with the same volume of methanol. The plates were sealed with ParaFilm, incubated at 30 °C, and monitored.

***S. coelicolor* M145 Co-culture Bioassay.** This procedure was inspired by a previously published *Streptomyces* co-culture assay (8). *S. coelicolor* M145 spore stocks were diluted to 3.33 x10^7 spores/mL, and 3 µL (1 x 10^5 spores) were spotted onto two SMMS plates and left to dry. *S. coelicolor* M1152::*pCAP01* and M1152::*aox* spore stocks were diluted to 1.33 x10^8 spores/mL, and 3 µL (4 x 10^5 spores) were spotted 2.0 cm away from *S. coelicolor* M145 and left to dry. The plates were wrapped in ParaFilm, incubated at 30 °C, and monitored.

***S. coelicolor* M1152 RNA Extraction and Gene Expression Analyses.** Confluent lawns of M1152::pCAP01 or M1152::*aox* spores (5 x 10^6) were spread onto cellophane discs on top of MS agar using water and let open to dry in a biosafety cabinet. The dried plates were sealed with ParaFilm and incubated at 30 °C until sporulation began, as indicated by the emergence of gray spores (about 4 days). The mycelium was scraped from the cellophane and ~70 mg was suspended in DNA/RNA Shield™ before being stored at -80 °C. Total RNA was isolated using the ZymoBIOMICS DNA/RNA Miniprep Kit with on-column DNAase treatment according to the manufacturer's instructions. Reverse transcriptase-quantitative PCR (RT-qPCR) analysis was conducted using the Luna® Universal One-Step RT-qPCR Kit Protocol according to the manufacturer's instructions. Oligonucleotide primers were used to specifically amplify target gene transcripts [*rpoB*-Fwd: CTTCGAGCCTCCCAAGAACA, *rpoB*-Rev: TTGGTCATGAGCGGGAAGT, *sco3201*-Fwd: AGGCCCTGTCCGACGAAGA, *sco3201*-Rev: TGAGCAGCACCATTCCAGCA, *hrdB*-Fwd: GCATGCTCTTCCTGGACCTCAT, *hrdB*-Rev: TGGAGAACTTGTAGCCCTTGGTGTA, *scbA*-Fwd: TGGAGGTGGAAGTGGCCTGT, *scbA*-Rev: TGAAGCGCGTCGTGGCAGT] (9–11). Fold change was calculated by the $2^{-\Delta\Delta Ct}$ method, with double normalization to housekeeping gene expression (*hrdB*) and the M1152::pCAP01 control. *scbA* expression is reported in Fig. S2A on Figshare at (https://doi.org/10.6084/m9.figshare.32564454) (12). Libraries for RNA-Seq were prepared and underwent ribosomal depletion then sequenced on the Illumina NextSeq 2000 platform with PE150 runs. The sequenced paired-end reads were trimmed using TrimGalore v0.6.10 at default settings and aligned to the *Streptomyces coelicolor* A3(2) reference genome (RefSeq assembly GCF_000203835.1) using the Bowtie 2 aligner. Differentially expressed genes were identified using the DESeq2 R package (v. 1.42.1).

Treatment with 1 and Secondary Metabolite Production. 2.5 µg of **1** was added to freshly inoculated 10 mL cultures and incubated as described above. After 7 days, the cultures were centrifuged at 6000 x*g* for 10 min at 4 °C, then the supernatant was decanted into a separate container and extracted with an equal volume of ethyl acetate. The remaining cell pellet was extracted with 10 mL of butanol with sonication. The extracts were combined, dried, and lyophilized. Once dry, the extracts were redissolved in 50 µL of methanol and analyzed by HR-ESI-MS. Representative secondary metabolites (cobaltribin, ascamycins, surugamides, and gamma-butyrolactones (GBLs)) were identified based on matching *m/z* exact masses and MS/MS fragmentation patterns with previously reported studies (13–16). Normalized peak areas were calculated by dividing the representative metabolite peak areas by the total peak area of the corresponding sample's total ion current (TIC). GBL production is reported in Fig. S2B on Figshare at (https://doi.org/10.6084/m9.figshare.32564454) (12).

Proposed Biosynthesis and Genome Mining. The *aox* BGC was identified and characterized by mining the *S. davaonensis* DSM 101723 genome (NCBI RefSeq assembly:

GCF_000349325.1) with antiSMASH 8.0 using relaxed strictness (17). Further domain analysis of AoxC was conducted using the web-based InterProScan service (18). Based on these annotations, **1**'s biosynthesis was proposed. In short, AoxC's CoA ligase (CAL) domain activates a fatty acid that is then condensed with an adenylated threonine and cyclized by the heterocyclization (Cy) domain. The McbC/SagB-like oxidoreductase (OR) domain (19) then oxidizes the (methyl)oxazoline into a (methyl)oxazole before offloading by AoxA and methylation by AoxB to form **1** (Fig. 2B). Homologs of *aox*-encoded proteins were first identified by a BLASTp search with the amino acid sequence of AoxC (20). The surrounding genes were identified by RODEO software and filtered based on the presence and adjacent proximity of *aoxA*, *aoxB*, and *aoxC* homologs (21). The resulting 61 BGCs were analyzed by antiSMASH 8.0 and their NRPS domain architectures manually curated, yielding 45 homologous BGCs. Adenylation domains with substrates that could not be predicted by antiSMASH were analyzed by the Predictive Algorithm for Resolving Adenylation domain Selectivity (PARAS) webtool (22).

Dataset S1 (separate file). NMR assignment data and spectra (^1H, ^{13}C, COSY, HSQC, HMBC) of **1** recorded in $CDCl_3$.

Dataset S2 (separate file). RT-qPCR C_q values and fold change calculations of M1152::*aox* and M1152::pCAP01.

Dataset S3 (separate file). RNA-Seq results of M1152::*aox* compared to M1152::pCAP01.

Dataset S4 (separate file). Peak areas of *Streptomyces* secondary metabolites and normalization calculations.

SI References

1. T. Kieser, M. J. Bibb, M. J. Buttner, K. F. Chater, D. A. Hopwood, *Practical streptomyces genetics* (The John Innes Foundation, 2000).
2. D. Xue, *et al.*, Discovery of acylsulfenic acid-featuring natural product sulfenicin and characterization of its biosynthesis. *Nat. Chem.* **17**, 1011–1019 (2025).
3. E. Takano, R. Chakraburtty, T. Nihira, Y. Yamada, M. J. Bibb, A complex role for the γ-butyrolactone SCB1 in regulating antibiotic production in *Streptomyces coelicolor* A3(2). *Mol. Microbiol.* **41**, 1015–1028 (2001).
4. K. Murai, Y. Takahara, T. Matsushita, H. Komatsu, H. Fujioka, Facile preparation of oxazole-4-carboxylates and 4-ketones from aldehydes using 3-oxazoline-4-carboxylates as intermediates. *Org. Lett.* **12**, 3456–3459 (2010).
5. M. Madden, *et al.* Figure S1: Growth-dependent production of compound **1**. Figshare. Available at: https://doi.org/10.6084/m9.figshare.32563290. Deposited 03 June 2026.
6. E. Takano, *et al.*, Purification and structural determination of SCB1, a γ-butyrolactone that elicits antibiotic production in *Streptomyces coelicolor* A3(2)*. *J. Biol. Chem.* **275**, 11010–11016 (2000).
7. N.-H. Hsiao, M. Gottelt, E. Takano, Chapter 6. Regulation of antibiotic production by bacterial hormones. *Methods Enzymol.* **458**, 143–157 (2009).
8. B. Bonet, *et al.*, The cvn8 conservon system is a global regulator of specialized metabolism in *Streptomyces coelicolor* during interspecies interactions. *mSystems* **6**, 10.1128/msystems.00281-21 (2021).
9. S. Honma, S. Ito, S. Yajima, Y. Sasaki, Nitric oxide signaling for actinorhodin production in Streptomyces coelicolor A3(2) via the DevS/R two-component system. *Appl. Environ. Microbiol.* **87**, e00480-21 (2021).
10. J. Zhang, *et al.*, The inhibition of antibiotic production in *Streptomyces coelicolor* over-expressing the TetR Regulator SCO3201 is correlated with changes in the lipidome of the strain. *Front. Microbiol.* **11** (2020).
11. A. Millan-Oropeza, C. Henry, C. Lejeune, M. David, M.-J. Virolle, Expression of genes of the Pho regulon is altered in *Streptomyces coelicolor*. *Sci. Rep.* **10**, 8492 (2020).
12. M. Madden, *et al.* Figure S2: Measurement of *S. coelicolor* GBL production. Figshare. Available at: https://doi.org/10.6084/m9.figshare.32564454. Deposited 03 June 2026.
13. D. Xue, *et al.*, Discovery and biosynthetic interrogation of a cobalt-containing terpene-polyketide-nonribosomal peptide tribrid with anticoronavirus activity. *J. Am. Chem. Soc.* **147**, 38951–38957 (2025).
14. C. Zhao, *et al.*, Characterization of biosynthetic genes of ascamycin/dealanylascamycin featuring a 5′-*O*-sulfonamide moiety in *Streptomyces* sp. JCM9888. *PLoS ONE* **9**, e114722 (2014).
15. Z. A. Maw, *et al.*, Discovery of acyl-surugamide A2 from marine *Streptomyces albidoflavus* RKJM-0023—a new cyclic nonribosomal peptide containing an N-ε-acetyl-L-lysine residue. *Molecules* **29** (2024).
16. J. D. Sidda, *et al.*, Overproduction and identification of butyrolactones SCB1–8 in the antibiotic production superhost *Streptomyces* M1152. *Org. Biomol. Chem.* **14**, 6390–6393 (2016).

17. K. Blin, *et al.*, antiSMASH 8.0: extended gene cluster detection capabilities and analyses of chemistry, enzymology, and regulation. *Nucleic Acids Res.* **53**, W32–W38 (2025).
18. P. Jones, *et al.*, InterProScan 5: genome-scale protein function classification. *Bioinformatics* **30**, 1236–1240 (2014).
19. E. Akiva, J. N. Copp, N. Tokuriki, P. C. Babbitt, Evolutionary and molecular foundations of multiple contemporary functions of the nitroreductase superfamily. *Proc. Natl. Acad. Sci. U.S.A.* **114**, E9549–E9558 (2017).
20. S. F. Altschul, W. Gish, W. Miller, E. W. Myers, D. J. Lipman, Basic local alignment search tool. *J. Mol. Biol.* **215**, 403–410 (1990).
21. J. I. Tietz, *et al.*, A new genome-mining tool redefines the lasso peptide biosynthetic landscape. *Nat. Chem. Biol.* **13**, 470–478 (2017).
22. B. R. Terlouw, *et al.*, PARAS: High-accuracy machine learning of substrate specificities in nonribosomal peptide synthetases. *JACS Au* **6**, 2315–2336 (2026).
